# Exceptional, naturally occurring HIV-1 control: Insight into a functional cure

**DOI:** 10.1016/j.medj.2024.06.008

**Published:** 2024-07-15

**Authors:** María Salgado, Stephen A. Migueles, Xu G. Yu, Javier Martinez-Picado

**Affiliations:** 1 IrsiCaixa Immunopathology Research Institute, 08916 Badalona, Spain; 2 CIBERINFEC, 28029 Madrid, Spain; 3 Germans Trias i Pujol Research Institute (IGTP), 08916 Badalona, Spain; 4 Laboratory of Immunoregulation, Division of Intramural Research, and Collaborative Clinical Research Branch, Division of Clinical Research, National Institute of Allergy and Infectious Diseases (NIAID), National Institutes of Health (NIH), Bethesda, MD, USA; 5 Ragon Institute of MGH, MIT, and Harvard, Cambridge, MA, USA; 6 Infectious Disease Division, Brigham and Women’s Hospital, Boston, MA, USA; 7 University of Vic – Central University of Catalonia (UVic-UCC), 08500 Vic, Spain; 8 Catalan Institution for Research and Advanced Studies (ICREA), 08010 Barcelona, Spain

## Abstract

Exceptional elite controllers represent an extremely rare group of people with HIV-1 (PWH) who exhibit spontaneous, high-level control of viral replication below the limits of detection in sensitive clinical monitoring assays and without disease progression in the absence of antiretroviral therapy for prolonged periods, frequently exceeding 25 years. Here, we discuss the different cases that have been reported in the scientific literature, their unique genetic, virological, and immunological characteristics, and their relevance as the best model for the functional cure of HIV-1.

## INTRODUCTION

In any infectious process, the interaction established between the pathogen and the host will determine the pathogenic process and its clinical severity. HIV-1 infection is no exception. The vast majority of cases with this lentiviral infection will progress inexorably along a standard disease course toward profound immunodeficiency. The development and implementation of antiretroviral therapy (ART) has resulted in successful pharmacologic control of viral replication and associated limitations on immunopathogenesis, which skew the balance of HIV infection in favor of the host.

In contrast to the majority of untreated people with HIV-1 (PWH), a small proportion (<1%), termed elite controllers (ECs), have maintained plasma viremia below detectable levels in sensitive monitoring assays and exhibited stable disease courses for many years in the absence of ART.^1–3^ Despite relatively low numbers and small cohort sizes, much has been discovered about the correlates of immune control associated with nonprogressive HIV-1 infection. However, these subjects have heterogeneous clinical outcomes, including a proportion that loses HIV-1 control over time.^4–6^

Among ECs exists an even rarer subgroup of extreme outliers. These exceptional ECs (EECs) exhibit higher-level control of virus replication compared to ECs, with very-difficult-to-recover plasma and cell-associated virus in the absence of ART for extended periods of time. Delving into the unique features that distinguish this group of EECs from other ECs has attracted the attention of several research groups for their potential contributions to understanding the underlying immunovirological mechanisms at work and also how to elicit long-term nonprogressive HIV infection. Indeed, it has been suggested that these extraordinary individuals represent the best model for functional HIV-1 cure.^7,8^

## DEFINING EECs

A consensus definition of EECs has not been established but should include PWH who exhibit spontaneous, high-level control of viral replication below the limits of detection in sensitive clinical monitoring assays (20–50 copies/mL) and no disease progression in the absence of ART for prolonged durations, frequently extending beyond 25 years. Other associated characteristics that support exceptionally limited virus replication and infrequent *in vivo* immune system stimulation by viral antigens include a difficult-to-detect HIV reservoir, HIV-specific cellular immune responses that are weak relative to those measured in ECs, and less reactive HIV-specific antibodies compared with other chronically infected PWH, suggesting partial seroreversion. Therefore, much interest has been addressed to characterize the HIV reservoirs of EECs, including quantification, genetic evolution, intactness, and genomic location. Likewise, host immunogenetics as well as HIV-specific immune responses have also been studied.

## CLINICAL EVOLUTION OF REPORTED EECs

**Figure 1.**
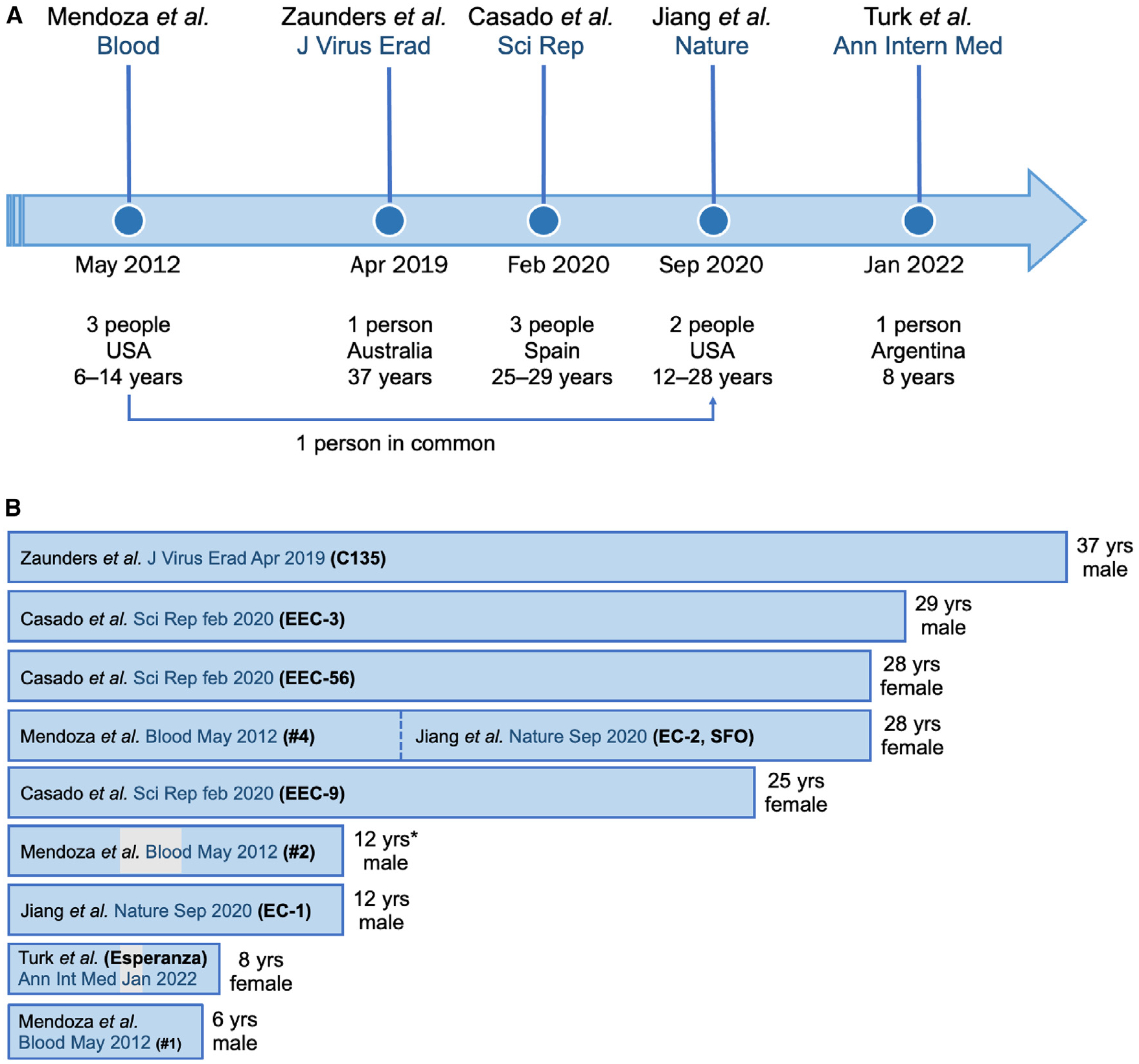
**EEC cases in people with HIV** (A) Timeline indicating the scientific articles that describe the cases of exceptional elite controllers (EECs) included in this perspective and their geographical origin. (B) Individual cases of EECs including code, time from diagnosis, and gender.

The published reports describing cases of EECs over the last 10 years have been limited in number yet comprehensive in scope in terms of scientific investigation (Figures 1A and 1B). The first report in 2012 included four cases, with at least three having a highly unique profile distinguishing them from other ECs.^9^ Subject C135, a member of the Sydney Blood Bank Cohort (SBBC) infected in 1981 with attenuated *nef*/3′ long terminal repeat (LTR)-deleted HIV-1 through transfusion from a common donor, was reported in 2019 as a case of very long-standing control and possible HIV-1 clearance 37 years after infection.^10^ Subsequently, five individuals (one reported previously) were described in two publications in 2020,^11,12^ and another EEC was reported in 2022.^13^ Case 4 from the first publication was EC-2 in a subsequent report and will be referred to herein as the San Francisco case. Interestingly, 56% of the described cases were women from developed countries, a percentage that appears to be higher than the expected prevalence if the distribution of EECs had been random in the whole PWH population. Remarkably, three female EEC cases had healthy pregnancies without vertical transmission, despite the absence of peripartum ART in two cases,^11^ and one additional case was transiently treated with ART during the third trimester of her pregnancy.^13^

The clinical follow-up of these case reports included a median of 24 (range 6–64) plasma viral load tests per person, which were below the limit of detection of contemporary assays except for two nonconsecutive “blips” of viremia below 400 HIV RNA copies/mL. When HIV RNA levels using ultrasensitive or single-copy assays were assessed in plasma samples, HIV RNA was below 0.4 copies/mL in all reported EECs except in one sample with 2 HIV RNA copies/mL, suggesting that residual viremia was a rare phenomenon in these cases. On the other hand, median absolute CD4^+^ T cells in the latest available determinations published for all the cases was 921 (range: 529–1,488), and the ratio of CD4/CD8 was greater than 1 except for the Esperanza case, in whom it was variable.^13^ These values are suggestive of normal T cell immunity, similar to people without HIV.

## HIV RESERVOIRS AND VIRAL FACTORS IN EECs

The HIV reservoir has been rigorously investigated in all EECs. Proviral DNA levels have been quantified in total peripheral blood mononuclear cells (PBMCs), total CD4^+^ T cells, or resting CD4^+^ T cells, with values below 30 copies of HIV per million cells, from analyses of up to 4 million total CD4^+^ T cells (Table 1). In the San Francisco patient’s case, the intact proviral DNA assay was negative when up to 14 million resting CD4^+^ T cells were analyzed. Quantitative viral outgrowth assay was performed in all cases as a measure of the persistence of infected cells with the capacity to be reactivated upon strong *in vitro* stimulation, leading to multiple cycles of viral replication in primary cell cultures. Despite variability in the number of tested cells, extending up to 560 million resting CD4^+^ T cells in the more thoroughly studied cases,^9,12^ no positive cultures for HIV replication were reported, with variable limits of quantification reaching as few as 0.004 infectious units per million cells (Table 1). Finally, in selected cases, HIV reservoirs in gastrointestinal tissues were also examined,^9,12^ with either extremely low or undetectable results (Table 1).

To contextualize these results, it should be noted that in PWH on suppressive ART, median levels of total HIV DNA in PBMCs range between 100 and 400 copies per million cells,^14–16^ which have been shown to be higher in lymphoid tissues, with median values of replication competent virus approaching 1 infectious unit per million cells.^17^ Altogether, these data suggest that the HIV reservoir in EECs is scarce, even in lymphoid tissue, and most likely represents defective or replication-incompetent viruses. The presence of “fossil” HIV nucleic acids has also been reported in PWH who have been reported to be cured of HIV after an allogeneic stem cell transplantation with CCR5Δ32/Δ32 donor cells, despite the absence of viral replication after several years of ART interruption.^18–20^

The development of new molecular techniques has facilitated near-full-length, single-genome sequencing of HIV. At least in five cases, this complex approach has been explored at different levels of depth, in some cases up to a billion PBMCs.^11–13^ Essentially, all of the individual viral sequences obtained showed deletions, hyper-mutations, or mutations in parts of the HIV genome, whose integrity is crucial for maintaining its transcription and translation into new infectious virions, such as nef/LTR^10^ or the major splice donor site.^11^ A recent report suggests that a distinct proviral landscape may be associated with more persistent control of HIV-1 without ART, with clonally expanded intact proviruses preferentially located in the heterochromatin.^21^

To also assess viral evolution and genetic variability, which might indirectly reflect present or past rounds of viral replication, longitudinal sequencing of highly variable viral genes has also been investigated over extended periods of time in peripheral cells of EECs (EEC3, EEC9, EEC56). The generated data point to very restricted genetic diversity (0.010 ± 0.003 synonymous versus nonsynonymous substitutions) suggesting null viral genetic evolution, which is up to 8-fold smaller than that observed in ECs and ART-suppressed PWH.^11^ Finally, some effort has also been deployed to study the envelope functionality of proviral sequences found in EECs. Cloned envelopes from EEC3, EEC9, and EEC56 showed ineffective *in vitro* binding to the CD4 receptor and the subsequent signaling activity to modify the actin/tubulin cytoskeleton.^22^ Globally, the data suggest that low fusion capacity of these envelopes leads to deficient cellular entry and infection competency.

Analyses of proviral sequences derived from the PBMCs of EECs suggest that low-fitness founder viruses might have led to the establishment of the EEC phenotype. However, this interpretation is challenged by, among other reasons, failed attempts, despite intensive efforts, to isolate and examine replication-competent HIV-1 from EECs. In contrast, autologous viruses have been recovered from many ECs, and the role played by viral factors in mediating durable control has been rigorously investigated. Some early reports of individuals with delayed or nonprogressive HIV-1 infection suggested that infection with defective or attenuated viruses was likely causal.^23,24,25^ However, with the notable exception of subject C135,^10^ many of these individuals experienced eventual disease progression. More recently, attenuated viruses have rarely been implicated as the cause of durable control in most ECs since their autologous viral isolates contained intact genetic sequences^26,27^ and were replication competent, fully pathogenic, and able to induce CD4^+^ T cell depletion in humanized mice.^26,28,29,30,31,32,33^ Perhaps more compellingly, discordant pairs, wherein HIV-1 from a progressor was transmitted to a person who ultimately became an EC, have been reported.^34,35^ These results suggest that the attenuated viral fitness that has been observed in some EC isolates in chronic infection is likely a consequence of immune-induced mutations and less likely than host factors to be the cause of the EC phenotype. However, it remains possible that the transmission of viruses with attenuated replication capacity in certain hosts might lead to the development of the EEC phenotype.

## INFLAMMATION, IMMUNOLOGICAL CHARACTERISTICS, AND HOST GENETICS OF EECs

Several inflammatory biomarkers in plasma have been studied in some EECs.^11^ In general, inflammatory markers are similar to those in uninfected people and lower than in PWH on ART. Humoral responses to specific HIV antigens have also been quantified. All plasma samples from EECs remained weakly reactive when assayed by western blot or ELISA. Responses were, in general, lower than those found in ECs but higher than those in uninfected people. CD4^+^ T cells derived from EECs^9,11^ have exhibited susceptibility to infection with both R5-tropic and X4-tropic HIV, suggesting that passive resistance to infection is not explanatory for this phenotype.

Host immunogenetic factors have been linked to clinical HIV disease progression.^2,36–40^ Approximately 65%–95% of ECs have been reported to bear a protective HLA class I B allele like 15, 27, 44, 57, or 58. In genome-wide association studies, HLA*B57/58 was most consistently associated with low viral loads and the EC phenotype.^39,41–43^ The link between overrepresentation of favorable HLA class I molecules and high-level HIV-1 control appears to lie within the HIV-specific CD8^+^ T cell response. Following natural infection or vaccination, CD8^+^ T cell responses restricted by protective class I proteins, especially B57, tend to be immunodominant and highly functional.^40,44–48^ The same phenomena have been observed in the rhesus macaque model of simian immunodeficiency virus (SIV) infection, in which macaques bearing the protective major histocompatibility complex (MHC) class I alleles Mamu B*08 and Mamu B*17 are more likely to exhibit durable SIV control that is abrogated by CD8^+^ T cell depletion.^49,50^ Highly functional HIV-specific CD8^+^ T cell responses have also been observed in ECs lacking protective alleles that are restricted by HLA class I proteins that have been categorized as neutral or deleterious with respect to disease progression.^51^ Despite these well-established associations, many questions remain. Protective HLA class I alleles are neither necessary nor sufficient for the EC phenotype: up to 35% of ECs with high-level control in some cohorts lack a favorable allele, and most HIV-1-infected individuals carrying them experience progressive disease.^40,41,52^ The precise mechanism(s) underlying how favorable HLA alleles predispose to the development of control and what factors determine which individuals with a protective allele will become an EC or progressor remain unclear. Strong HIV-specific CD8^+^ T cell responses appear to be the mediators of this control, but it remains possible that other immune cells in the innate or adaptive immune system might also be involved. Nonetheless, it is striking that all 9 EECs carry at least one host genetic determinant of protection, including protective HLA class I B alleles (7/9 are B*57/58+) and polymorphisms affecting the expression of HLA C (Table 2). Compared to other recipients and the donor in the SBBC, C135 was positive for HLA B*57 as well as a class II allele that has been shown to restrict potent CD4^+^ T cell responses in the gut mucosa.^10,53,54^

HIV-specific T cell responses have been assessed in EECs.^9–11,13^ With PBMCs from subject C135, increased proliferation of HIV-specific CD4^+^ T cells to HIV peptides was noted. While this could represent one mechanism contributing to his EEC status,^53–55^ alternatively, this result might simply be a consequence of reduced viremia, as demonstrated in ART-suppressed progressors who exhibited improved CD4^+^ T cell proliferative responses to levels observed in ECs, even though their intrinsic capacity to restrict virus replication had not been enhanced.^56^ Frequencies of cytokine-secreting HIV-specific CD8^+^ T cells were higher than in HIV-negative individuals and ART-suppressed PWH, supporting that virus-specific T cell responses had been primed in EECs following HIV infection, but were relatively low compared to other chronically infected, untreated PWH.^9–11,13^ Following more prolonged stimulation, HIV-specific CD8^+^ T cells expanded significantly in some EECs, a feature that has been associated with spontaneous immune control.^9^ Cytotoxic capacity, measured by the ability of CD8^+^ T cells to eliminate HIV-infected CD4^+^ T cell targets or suppress p24 production ex vivo, overlapped with both ECs and viremic progressors.^9–11^ These responses were therefore fairly high in the setting of remarkably low antigen levels and remote antigen reencounter. T cell responses were also found to be polyfunctional comparable to other long-term nonprogressors/ECs but higher than those of ART-suppressed PWH.^11^ These results, paired with the strong association of protective HLA class I B alleles in all EECs, suggested that robust T cell responses, which might have waned due to very remote antigenic stimulation *in vivo*, could have contributed to the induction of this remarkable phenotype. These findings are reminiscent of the interesting outcome in rhesus macaques with protective MHC class I alleles immunized with live attenuated, *nef*-deleted SIV. Following challenge with heterologous strains of SIV, near complete control of acute phase viral replication and a rapid increase in virus replication after *in vivo* depletion of CD8^+^ T cells were observed, implicating MHC-class-I-restricted CD8^+^ T cells as playing a major role.^57^ Although the lines of evidence strongly suggest that functional HIV-specific CD8^+^ T cell responses are not merely a consequence of reduced viremia but play a major role in the development of the EC and EEC phenotypes, formal proof of causality is lacking.

To date, the number of reported HIV cured cases after an intended clinical intervention is very limited. While allogeneic hematopoietic stem cell transplant with CCR5Δ32/Δ32 donor cells is the most well-described strategy, with 5 published cases ranging from 12 to 1.5 years after analytical treatment interruption,^19,20,58,59,60^ other cases have also been reported: one autologous transplant of CCR5-edited CD4^+^ T cells with 6 years of control^61^; one case of immune therapy with a broadly neutralizing antibody plus a latency reversing agent administered shortly after antiretroviral initiation, with 4 years of control^62^; and two cases of early ART to avoid perinatal HIV transmission who have lived off therapy for 10 and 19 years, respectively.^63,64^ These cases share with EECs at least some virological and immunological characteristics that suggest a durable absence of virus replication despite the lack of ART.

## FUTURE PERSPECTIVES

Given the rarity of EECs and the tremendous potential they hold for uncovering cures for HIV infection, it will be essential to coordinate collaborative efforts and secure long-term funding opportunities to establish a larger, ideally unified international cohort of ECC cases. This initiative should facilitate the discovery of common biological mechanisms and biomarkers, as well as unique characteristics, among individuals. A key challenge lies in accessing historical samples, which may be feasible in certain instances due to the diligent efforts of research teams worldwide that have been systematically collecting biological specimens from PWH and may unknowingly possess valuable EEC samples in their repositories. It is important to highlight that within the framework of universal HIV treatment, new EEC cases may go unnoticed, which poses a challenge for their identification.

There are two remarkable aspects that also need investigation. The first relates to the possible existence of EECs across different human populations and HIV subtypes. The cases described so far have predominantly involved White individuals and subtype B viruses. However, it is plausible that this phenotype may also be present in other human populations and viral subtypes, which would warrant further study for comprehension and provide optimism that exceptional control could occur in diverse host-virus scenarios.

Secondly, the increased proportion of women among the described EEC cases suggests there may be a potential sex-associated influence on triggering or sustaining the phenotype. There is a growing understanding of the role that sex-based differences play in HIV acquisition, pathogenesis, and treatment response.^65^ Nevertheless, there are still significant gaps in comprehending the impact of biological sex on the viral reservoir and the implications of these variances in developing potential HIV-1 curative strategies.^66^ Both population-based disparities and sex-based differences could stem from exclusive host-dependent phenomena or host-pathogen interactions that contribute to the extreme restriction of viral replication observed in EEC cases.^67^

The presence of HIV proviruses that are unable to replicate in these PWH suggests that studying EECs may offer biologically intriguing insights for novel approaches recently developed to permanently silence viral transcription. This can be achieved through disrupting proviruses from host cells via gene editing, utilizing potential latency-promoting agents to block and lock integrated viral genomes, or employing viral RNA interference strategies.^68^ Alternatively, the association of exceptional control with host immunogenetics and the higher-than-expected frequencies of virus-specific T cell responses with high killing potential suggests that further investigation of EECs could provide deeper understanding of highly effective antiviral effector mechanisms that predict exceptional viral control (or functional cure), contribute to the design of efficacious vaccines and immune-based therapies, and/or inform shock-and-kill cure strategies to purge the HIV reservoir.

## CONCLUSIONS

**Figure 2.**
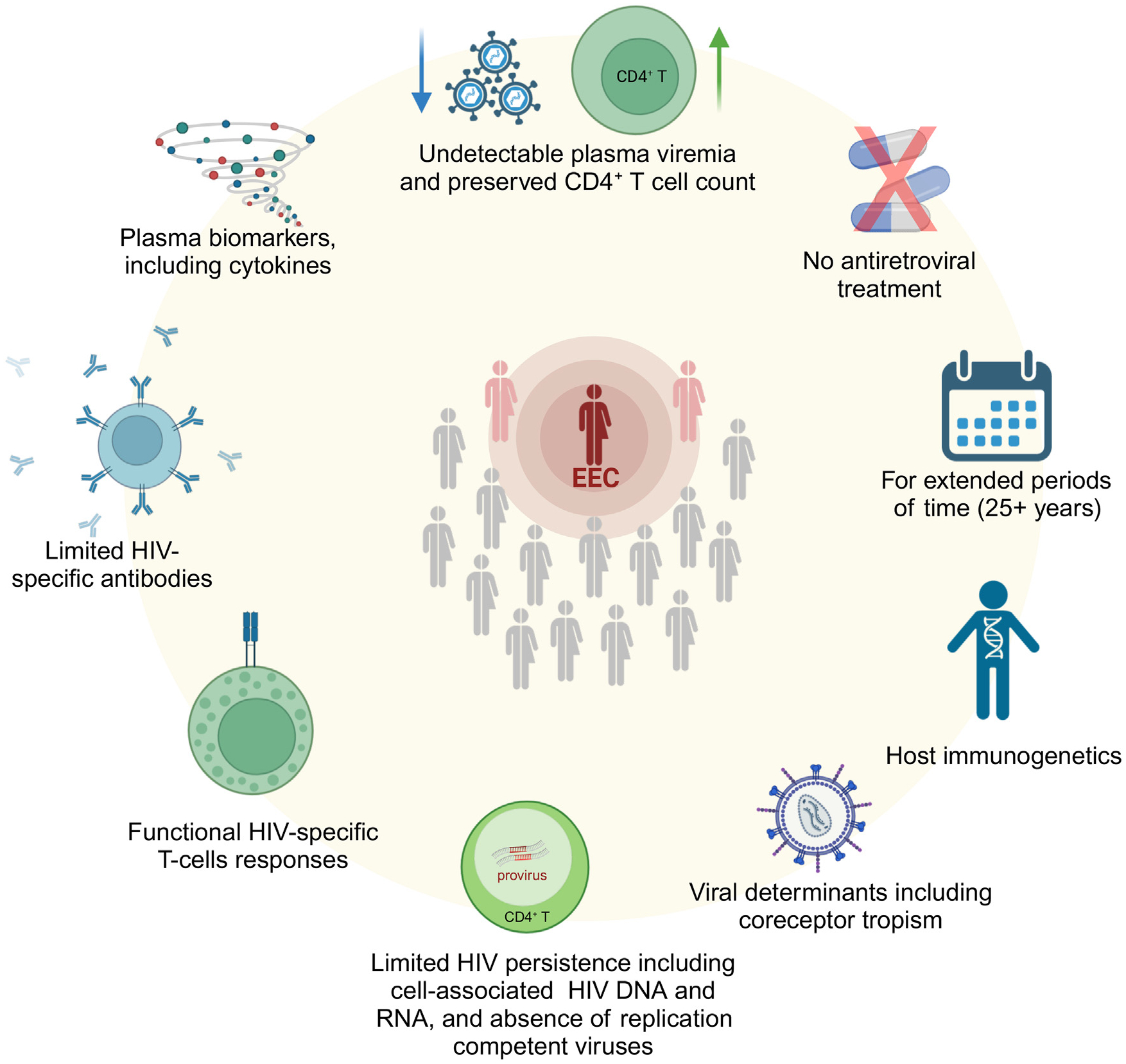
Clinical, virological, and immunological characteristics of EECs in people with HIV, who represent a unique and valuable model for studying a functional cure for HIV. Generated with BioRender

EECs harbor a remarkably low, and apparently defective and replication-incompetent, viral DNA reservoir, with practically null viral genetic evolution and extremely low complexity of the viral populations. Low viral population size and viral diversity are associated with low viral fitness, which would be consistent with the absence of viral replication for periods that might exceed 25 years. Further contribution of host genetic factors and HIV-specific adaptive immune responses might also have played a role in the outcome of this clinical phenotype. Difficult-to-detect proviral reservoirs, lack of reservoir evolution, and weakly reactive, nonevolving HIV-specific antibody response profiles suggest that HIV-1 replication was restricted very early after infection. The features mentioned above, along with very low levels of immune activation, would support the idea that EECs provide evidence that nearly complete suppression of HIV-1 replication is possible in humans and, as such, represents the best model for a functional cure of HIV (Figure 2). Immediate ART upon HIV infection diagnosis can definitively encrypt cases of spontaneous HIV control. At least some of the characteristics described in EECs might help identify cases among treated individuals, including a very low reservoir level.^14,69^ Upon the publication of some of the articles reported in this perspective and presentations at international conferences, new cases of EECs from several clinics in different countries have been communicated to the authors, some now exceeding 30 years since diagnosis, and probably more from primary infection. Expanding currently studied cases might help better define the immune-virological phenotype of EECs.

Pairing observations in EECs with the large body of investigation on ECs, it is tempting to hypothesize that primary infection in EECs might have occurred with a low-fitness viral founder strain or that initial innate/intrinsic immune responses might have shaped the selection of an unfit virus. We have summarized evidence of a strong association in EECs with favorable host immunogenetic factors, with 7/9 (78%) bearing the most protective of HLA class I alleles B*57/58, and relatively strong HIV-specific CD8^+^ T cell responses possessing increased cytolytic potential. While extreme attenuation of the infecting founder virus or higher immune responsiveness relative to ECs could yield the EEC phenotype, these findings in aggregate, considered along with outcomes in C135 and the SBBC and highlighted reports of SIV infection in rhesus macaques, support a model in which an attenuated, low-fitness founder virus and robust early host immune responses together are likely required for exceptional HIV-1 control. As such, EECs probably are distinct from most ECs and not merely at the extreme end of the disease progression/controller spectrum. It is probably too premature to speculate whether, in view of these EECs, it would be possible to design a medical intervention to induce a permanent control of HIV-1 pathogenesis. Nonetheless, the extremely long-term HIV control in the absence of ART makes this group of PWH outstanding examples of exceptional virus-host interactions that could provide broader insight relevant to understanding the immunopathogenesis of other current or future retroviruses, coronaviruses, and other pathogens with extensive genetic diversity and formidable immune-evasive properties.

## Figures and Tables

**Table 1.** Proviral reservoir and replication-competent proviruses in blood and tissue samples

Case	HIV DNA (copies/10^6^ cells), cells analyzed	qVOA (IUPM/10^6^ cells), cells analyzed	Tissues (HIV DNA copies/10^6^ cells), cells analyzed
C135	HIV DNA^neg^, in 11.2 × 10^6^ PBMCs and in 2 × 10^6^ memory CD4^+^	N/A	HIV DNA^neg^, in 0.16 × 10^6^ CD4^+^ from gut and in 0.016 × 10^6^ CD4^+^ from lymph node
EEC-3	27.09, in 4 × 10^6^ tCD4^+^	<0.025, in 28 × 10^6^ tCD4^+^	N/A
EEC-56	10.05, in 3.2 × 10^6^ tCD4^+^	<0.018, in 63 × 10^6^ tCD4^+^	N/A
#4/SFO, EEC-3	IPDA^neg^, in 14 × 10^6^ rCD4^+^	<0.004, in 41 × 10^6^ tCD4^+^ and in 340 × 10^6^ rCD4^+^	HIV DNA^neg^, in 4 × 10^6^ CD45^+^ from rectum and ileum^a^
EEC-9	8.75, in 2.6 × 0^6^ tCD4^+^	<0.018, in 38 × 10^6^ tCD4^+^	N/A
#2	25.2, in PBMCs	<0.002, in 560 × 10^6^ tCD4^+^	2.8, in colon CD4 cells
EC-1	0.02162, in 1,020 × 10^6^ tCD4^+^	<LOD, in 30 × 10^6^ tCD4^+^	N/A
Esperanza	N/A	<LOD, in 150 × 10^6^ rCD4^+^	placenta (neg)
#1	6.56, in PBMCs	<0.004, in 240 × 10^6^ tCD4^+^	N/A

PBMCs, peripheral blood mononuclear cells; IPDA, intact proviral DNA assay; rCD4^+^, resting CD4^+^; tCD4^+^, total CD4^+^; qVOA, quantitative viral outgrowth assay; IUPM, infectious units per million cells; <LOD, below the limit of detection; neg, negative; N/A, not available.

a A previous sample from 2012: <2.6 copies/10^6^ cells in colon and 42.4 copies/10^6^ cells in ileum.

**Table 2.** Immunogenetic markers associated with HIV disease progression

Case	CCR5	HLA class I, A loci	HLA class I, B loci	Other reported genetic factors
C135	WT/Δ32	01, 33	50, 57^a^	*nef*/LTR-deleted, HLA-DR13
EEC-3	WT/WT	02, 02	27,^a^ 58^a^	HLA C (rs9264942), −35 TT→CC
EEC-56	WT/WT	01, 02	14, 57^a^	HLA C (rs9264942), −35 TT→CC
#4/SFO	WT/WT	02, 30	13, 57^a^	–
EEC-9	WT/WT	02, 31	39, 57^a^	HLA C rs9264942, −35 TT→CC
#2	WT/WT	–	15,^a^ 57^a^	–
EC-1	WT/WT	01, 68	55, 57^a^	–
Esperanza	WT/WT	02, 31	15,^a^ 44^a^	–
#1	WT/D32	24, 31	40, 44^a^	–

WT, wild type.

a HLA alleles associated with HIV disease protection.
